# Lessons from a peer-led obesity prevention programme in English schools

**DOI:** 10.1093/heapro/dau008

**Published:** 2014-04-05

**Authors:** Sarah L. Bell, Suzanne Audrey, Ashley R. Cooper, Sian Noble, Rona Campbell

**Affiliations:** 1 School of Social and Community Medicine, University of Bristol, BS8 2PS, UK; 2 Centre for Exercise, Nutrition and Health Sciences, University of Bristol, BS8 1TZ, UK

**Keywords:** school based, adolescent, obesity prevention, health education

## Abstract

Obesity in young people is a major public health concern. Energy balance, the interrelationship between diet and physical activity, is known to be a key determinant. Evidence supports the development of school-based approaches to obesity prevention. ASSIST (A Stop Smoking in Schools Trial) is an effective school-based, peer-led smoking prevention programme for 12–13-year-old students, based on diffusion of innovations theory. The AHEAD (Activity and Healthy Eating in ADolescence) study tested the feasibility of adapting ASSIST to an obesity prevention intervention. The AHEAD intervention was tested and refined during a pilot study in one school, followed by an exploratory trial in six schools. Quantitative (self-report behavioural questionnaires and evaluation forms) and qualitative (structured observations, focus groups and interviews) research methods were used to examine the implementation and acceptability of the intervention. The potential effectiveness of the intervention in increasing healthy eating was measured using self-report behavioural questionnaires. Activity monitors (accelerometers) were used to measure physical activity. Results show it was feasible to implement the AHEAD intervention, which was well received. However, implementation was resource and labour intensive and relatively expensive. Furthermore, there was no evidence of promise that the intervention would increase physical activity or healthy eating in adolescents. Although diet and physical activity are both relevant for obesity prevention, the focus on two behaviours appeared too complex for informal diffusion through peer networks. This identifies a tension, particularly for adolescent peer-led health promotion, between the desire not to isolate or oversimplify health behaviours and the need to present clear, succinct health promotion messages.

## BACKGROUND

Childhood obesity is a major public health concern (WHO, 2012) associated with a range of health problems including adverse metabolic and cardiovascular conditions (Lawlor *et al.*, 2005). Over the past three decades the prevalence of overweight and obesity in young people has increased substantially (WHO, 2011) and globally 170 million children are now estimated to be overweight (Lobstein *et al.*, 2004). Energy balance, the interrelationship between diet and physical activity, is known to be a key determinant (Hill, 2006).

A Cochrane systematic review identified 55 controlled childhood obesity prevention studies published up to March 2010: 37 were included in the meta-analysis which demonstrated that programmes were effective in reducing adiposity (Waters *et al.*, 2011). However, not all individual interventions were effective, there was a high level of observed heterogeneity and small study bias was likely. It was therefore concluded that although there was evidence to support beneficial effects of these programmes, the findings must be interpreted cautiously.

Schools are considered conducive to promoting health behaviour due to there being sustained access to a target group over several years, and recent systematic review findings provide some encouragement that they are a good setting for obesity prevention. Khambalia *et al.* (Khambalia *et al.*, 2012) synthesised existing systematic reviews and meta-analyses of school-based behavioural interventions for controlling and preventing obesity. Results from their review of reviews indicate that while studies are heterogeneous, there are certain intervention components in the school setting associated with a significant reduction of weight in children. These include combined diet and physical activity interventions, interventions that include a family component and long-term, as opposed to short-term, interventions.

### The ASSIST model

The influence of peers on young people's health behaviours during adolescence is acknowledged (Maxwell, 2002; Steinberg and Monahan, 2007; Valente *et al.*, 2013) and interventions using a peer-led teaching model for health promotion have shown positive effects (Harden *et al.*, 1999). ASSIST (A Stop Smoking in Schools Trial) evaluated a school-based peer-led smoking prevention intervention that was shown to be effective in reducing smoking uptake (Campbell *et al.*, 2008). The ASSIST intervention was informed by diffusion of innovations theory which argues that behaviour change is initially propelled by ‘early adopters’ who are often popular or well-regarded individuals (Rogers, 1983). During ASSIST, all Year 8 students (aged 12–13 years) in participating schools were asked to complete a questionnaire to identify influential students in the year group (Starkey *et al.*, 2005). These potential ‘early adopters’ were invited to train as ‘peer supporters’ to intervene in everyday situations and encourage their peers not to smoke. The aim was to recruit at least 15% of the year group to diffuse the health promotion message (Kelly, 2004). Peer supporters were given 2 days of knowledge- and skills-based training away from school, provided by specialist trainers, and received four further follow-up sessions in school over the subsequent 10-week intervention period to support and encourage them in their role (Audrey *et al.*, 2004). Evidence from the ASSIST process evaluation suggested that the peer supporter recruitment process was a strength of the ASSIST model. The peer supporters, working informally rather than under the supervision of teaching staff, engaged sufficiently with the task they were asked to undertake to be effective in diffusing health-promotion messages to their peers (Audrey *et al.*, 2006). Furthermore, the intervention was acceptable to schools and teaching staff (Audrey *et al.*, 2008).

Given the success of ASSIST, the aim of the AHEAD (Activity and Healthy Eating in ADolescence) study was to test the feasibility of adapting the ASSIST intervention to increase physical activity and healthy eating in adolescents. Physical activity levels are known to decline with age in both sexes, although more steeply in girls (Department of Health, 2011) and adolescents become more autonomous in their eating behaviours (Story *et al.*, 2002). Furthermore, behaviour patterns acquired during this period are likely to influence long-term behaviours (Centers for Disease Control and Prevention, 1996). In this paper we describe the development, implementation and acceptability of the AHEAD intervention. We identify important differences between ASSIST and AHEAD with implications for the effectiveness of the AHEAD intervention.

## METHODS

### Study design

The AHEAD study incorporated Phase I and Phase II of the Medical Research Council's framework for evaluating complex interventions (MRC, 2000, 2008). The intervention was tested and refined during a pilot study in one school followed by an exploratory trial in six schools (three randomized to receive the intervention and three in the control arm). The study included a process evaluation to examine the context, development, implementation and acceptability of the intervention, and an assessment of costs.

### Research governance

Ethical approval was obtained from the University of Bristol Faculty of Medicine and Dentistry Research Ethics Committee. Written consent was provided by the head teacher for the schools' participation in the study. Parents of students selected to attend the training provided written consent, and the participating students then assented to take on the role of a peer supporter.

### Developing the AHEAD intervention

Six focus groups were conducted with young people to explore their views about physical activity and healthy eating: two in youth work settings and four with Year 8 students (aged 12–13 years) in the pilot school. The school's catering manager and ‘Healthy Schools’ coordinator were also interviewed.

An ‘Intervention Development Group’ was formed which included members of the main research team and external consultants with specific expertise in adolescent health, physical activity, healthy eating and working with young people. A range of key policy, practice and research documents were consulted including the Foresight report on tackling obesity (Butland *et al.*, 2007). National policy documents (DfEE, 1999a, 1999b) were also examined to ensure that the AHEAD intervention complemented the school curriculum. This developmental work informed the AHEAD intervention that was implemented, tested and refined throughout Phases I and II.

### Setting

The pilot study was conducted in an inner-city state-funded comprehensive school, purposively selected because its student population had a broad range of backgrounds and educational needs. This enabled the acceptability of the intervention to be tested with a variety of young people. The exploratory trial was undertaken in six co-educational comprehensive schools.

### Trainers

Trainers were from an experienced training company specializing in health and well-being that also worked on the ASSIST study, and other trainers were recruited from a nutrition, health and exercise masters degree course at the university (a qualified teacher, a specialist in nutrition and a childhood obesity physical activity specialist). There were two lead trainers and one support trainer recruited per session. A key contact teacher from each intervention school attended the training with the group of peer supporters but was not involved in delivering the training.

### Stages and content of the AHEAD intervention

The six stages of the intervention, which replicated those in ASSIST, are described in Table 1. The AHEAD training programme for peer supporters aimed to increase knowledge and skills, and influence behaviour. The key messages in relation to physical activity were to increase the volume of physical activity and the amount of moderate-to-vigorous physical activity and to decrease the time spent sedentary. In relation to healthy eating the key messages were to increase breakfast consumption and fruit and vegetable intake, and reduce consumption of fizzy drinks, sugar, salt and fat.

**Table 1:** Stages of the AHEAD intervention

Training the trainers	All trainers involved in delivering the programme attend a training event to: become familiar with the training programme; practise sessions; encourage teamwork; consider health and safety issues
Peer nomination	A peer nomination questionnaire is completed by all Year 8 students to identify influential peers. Responses tallied to obtain a score for each student. ≥18% of year group with most nominations (gender balanced) invited to recruitment meeting
Peer supporter recruitment	Meeting with nominees to explain the intervention and the role of peer supporter and invite them to attend the training. Parental consent for training sought at this stage
Training	Two-day out of school training event focusing on the knowledge, skills and confidence to informally promote physical activity and healthy eating amongst other students in their school year group and to model such behaviour through adopting small changes in their own physical activity levels and diet as appropriate Participants who agree to take on the role of peer supporter sign an assent form Peer supporters receive a diary to record relevant interactions with their peers. The diaries include additional information and some ‘healthy challenges’ to encourage achievable changes in the peer supporters' behaviour
Support ‘follow-up sessions’	Four school-based follow-up sessions to support and encourage peer supporters in their role and provide further information about the benefits of healthy eating and physical activity
Acknowledgement	Certificates presented to all peer supporters who completed the 2-day training. £10 gift vouchers presented to those who continued in the role of peer supporter

Sessions were practical and interactive, and used a variety of delivery methods including drama, food preparation, information technology and games. Although there was a structured sequence of activities, trainers were given some flexibility over the pace and content to suit the needs and abilities of different groups. Activities were highlighted in the training manual as ‘essential elements’ or ‘optional extension activities’ to allow for this differentiation. Examples of activities, with associated learning objectives, are outlined in Table 2.

**Table 2:** Content of the AHEAD intervention: example activities

Objective	Activity
Training To understand the nutrient content and ingredients in processed and fast food and make a homemade burger	‘Ready, steady, cook’. Demonstration used a fast food burger recipe to illustrate poor quality of ‘fast food’. Peer supporters then made their own burger for lunch (meat or vegetarian) to demonstrate the difference in ingredients
To taste different fruit and vegetables	‘Taste trial’. Fresh fruit and vegetables were plated. Peer supporter volunteers were blindfolded and asked to guess the name of the fruit or vegetable they had tried, and describe the taste and texture
To emphasize the range of physical activity options	‘A-Z’. Peer supporters were asked to think of a physical activity beginning with each letter of the alphabet
To understand what sedentary behaviour is and its health implications	‘Before and after’ role plays. Sedentary behaviour (before) changed to active behaviour (after), e.g. taking the lift changed to walking the stairs
To consider barriers to physical activity and think of solutions	‘Barrier wall’. A wall of cardboard box ‘bricks’ was constructed. Peer supporters were asked to think of barriers to physical activity, which were written onto the bricks. Peer supporters who thought of solutions to barriers were allowed to remove the relevant brick until the wall was demolished
To develop communication skills for their role	‘Role play’. Peer supporters improvised conversations with their peers based on key messages about physical activity or healthy eating
To use goal setting to change health behaviour	‘Goal setting’. Peer supporters set themselves a small challenge in relation to physical activity or healthy eating. This was revisited at the follow-up sessions
Support ‘follow-up sessions’	
To understand the salt, sugar, fat and fibre content of various foods	A game of top trump cards. Cards display different food products. The winning card has the ‘healthiest’ level of a selected ingredient, e.g. salt, sugar, fat or fibre
To remind peer supporters of the importance of breakfast	A morning session with a discussion about how to make healthy choices at breakfast during which volunteers serve breakfast to their peers
To evaluate health promotion posters	Peer supporters complete jigsaws of current health promotion posters relating to physical activity and healthy eating, and discuss which they consider would have the greatest impact on their peers
To experience physical activity	Physical activity sessions including skipping, circus skills and Frisbee

Because the intervention was implemented by external trainers, there was a relatively low demand on the teachers' time. The training programme supported content in the school curriculum and other initiatives such as the National Healthy Schools Programme.

There were two important modifications to the practical arrangements for the AHEAD training when compared with ASSIST. The AHEAD peer supporters walked to the training venue to demonstrate ‘active travel’, whereas coaches were used to transport peer supporters during the ASSIST study. Secondly, unlike ASSIST, food and drinks for AHEAD were prepared on-site by the training team with the assistance of peer supporters where appropriate. This enabled more nutritious food to be provided and allowed the young people to be actively involved in the preparation of healthy drinks, snacks and meals.

### Examining the implementation and acceptability of the AHEAD intervention

During the exploratory trial, the implementation and acceptability of the intervention were examined using a mixture of quantitative and qualitative methods. The research and training teams completed evaluation forms at each stage of the intervention. Structured observations of all training activities were undertaken by the research team, and the peer supporters and teachers completed evaluation forms. Two post-intervention focus groups with peer supporters (*n* = 17) were conducted and digitally recorded to explore their views about each stage of the intervention.

Because the peer supporters were asked to informally diffuse the health promotion messages, it was not possible to observe whether they actively engaged in conversations about healthy eating and physical activity with their peers or modelled healthier behaviours with them. However, the post-intervention behavioural questionnaires in the intervention schools included questions to assess the diffusion of the messages. Additionally, two post-intervention focus groups were conducted with non-peer-supporters (*n* = 16) at which they were asked about the activities of the peer supporters.

Descriptive statistics were compiled in relation to the peer supporter recruitment and retention rates. Responses to the behavioural questionnaires were entered into an Access database. Notes from the structured observations were examined and responses in the evaluation forms were collated and summarized. Focus group recordings were fully transcribed and the textual data were scrutinized for differences and similarities within emerging themes, keeping in mind the context in which these arose.

As part of the evaluation, the cost of delivering the intervention, including the initial training of the trainers was assessed. Weekly timesheets and travel claim forms were completed by the trainers. All resource expenditure (including venue hire, external lead trainers, training materials and refreshments) was recorded and an account was made of the resources deployed at each stage of the intervention in each school.

### Examining the potential effectiveness of the AHEAD intervention

The diet and physical activity outcomes were measured at baseline and follow-up (7 months after baseline; ideally the time period between measures would have been longer but the trial needed to be completed within 1 academic year). These were objectively measured volume of physical activity (mean counts per minute) using accelerometers, and; self-reported target food consumption (frequency of target foods usually consumed, Table 3) using behavioural questionnaires completed by all the Year 8 students in the study schools. Minutes per day in moderate-to-vigorous physical activity (MVPA) and minutes per day of sedentary time were also measured objectively. For accelerometer data to be considered valid, and therefore included in analyses, students were required to wear the monitor for at least 10-h each day (600 min) on 3 or more days. Evenson *et al.*'s (Evenson *et al.*, 2008) actigraph cut-points, recommended by Trost *et al.* (Trost *et al.*, 2011), were used to determine minutes per day in MVPA and minutes per day of sedentary time (accelerometer processing decision details are available from the corresponding author).

**Table 3:** Results: target food consumption responses

Students who consume …	Control baseline	Intervention baseline	Control follow-up	Intervention follow-up
Breakfast either most days or every day	84% (373/445)	82% (363/441)	75% (323/431)	83% (361/435)
At least three portions of fruit a day	55% (242/438)	57% (251/439)	53% (223/422)	59% (257/433)
At least three portions of vegetables a day	56% (238/426)	58% (251/435)	57% (240/422)	57% (248/432)
Fizzy drinks more than once a day	25% (102/409)	21% (89/426)	18% (78/430)	18% (79/430)
Chocolate/biscuits/cakes more than once a day	25% (105/413)	17% (73/425)	18% (78/425)	16% (67/430)
Crisps/salty snacks more than once a day	19% (79/411)	12% (51/428)	14% (59/429)	12% (50/430)

## RESULTS

### Study participants

The pilot study was conducted with 99 Year 8 students of whom 19 (19%) trained as peer supporters. During the exploratory trial there were 928 Year 8 students across the 6 participating schools at baseline of which 462 students were in the intervention arm.

### Exploratory trial peer supporter recruitment and retention

In the intervention schools, the peer nomination stage was successfully implemented with 17% of the year group being recruited and trained as peer supporters (79 of the 462 students in the intervention arm at baseline). Attendance at school-based follow-up sessions ranged from 81 to 98%. This represented ≥16% of the year group across the three intervention schools for the first three sessions and 14% at the final follow-up session.

### Implementation and acceptability of the AHEAD intervention

Schools were able to organize the recruitment meeting and release the nominated peer supporters from school for the 2-day training event on dates negotiated between the school and the trainers. All school-based follow-up sessions were completed and no school withdrew from the study.

Teachers recorded favourable comments in their evaluation forms, including: ‘Healthy eating was valuable’ (School 21, contact teacher) and ‘Activities varied and a good mix of physical and mental’ (School 24, contact teacher). However, there was some evidence of teachers' concerns that the behavioural standards expected by the trainers were more relaxed than those enforced in the school setting: ‘Communication from trainers to pupils was at times not clear. You must establish ‘quiet’ in order to give out instructions’ (School 21, contact teacher).

### The training programme

Trainers commented that the ‘training the trainers’ sessions were valuable as they ensured familiarity with the intervention and built good relations amongst the team. Thereafter, the training programme was successfully delivered in all intervention schools. Overall, the trainers concluded that it was feasible to implement the AHEAD intervention but some concerns were raised. First, the programme included a large amount of information about both physical activity and healthy eating. The notes from the structured observations indicated that the programme was very full and there was little time to explain and consolidate knowledge. This was challenging for the trainers and prevented opportunities for the peer supporters to understand topics in any depth. Comments from evaluation forms included: ‘Quite a lot of content that made it hard to digest’ (School 22, trainer 3); ‘I need more help with thinking things through’ (School 22, peer supporter).

In addition, the training programme was labour and resource intensive. The purchase, storage and transportation of numerous resources (including food preparation equipment such as blenders and grills, physical activity equipment and assorted posters) required the use of a car or van and the need to build in extra time at the venues to unload and set-up the equipment. Health and safety was an important consideration for the food component: knives were used for chopping onions when making burgers, and allergies had to be considered during ‘taste trials’. In addition, a trainer with an appropriate food hygiene qualification was required to oversee all of the food preparation for activities and meal breaks.

Finding suitable training venues was a challenge. The requirements included: an environment that was accepting of potentially boisterous young people; adequate space for indoor physical activity; permission to use cooking facilities and within safe walking distance to the school (no further than a 30 min walk along a pedestrian-friendly route). Escorting the students along busy roads from school to the training venue required a high trainer:student ratio and diligence *en route*. The most relaxed and accessible venues were local sports or social clubs but considerable advanced planning was necessary to allow time to discuss requirements and negotiate with facility managers.

It was evident from observations that the peer supporters engaged with and enjoyed many of the activities and the comments from the trainers' and peer supporters' evaluation forms support this finding: ‘They were very involved and responded well to hands-on activities and role plays’ (School 22, trainer 6); ‘Excited, interested, loved it and no doubt got a lot from it’ (School 24, trainer 4); ‘I really enjoyed the food and smoothie making, I like fruit a lot more now’ (School 21, peer supporter); ‘The two days were grrrr-eat’ (School 24, peer supporter). The activities most enjoyed by the peer supporters were building and breaking down a physical activity barrier wall; role plays; games; a fruit and vegetable taste trial and making their own burgers (Table 2). Peer supporters were also asked if there were sessions they did not enjoy. None were given although it was suggested that the training could be improved by having even more games and less writing.

### School-based follow-up sessions

The evaluation forms completed by peer supporters after the follow-up sessions in schools also indicated that games were popular: ‘Didn't just sit down and did lots of activities’ (School 24, peer supporter). The peers supporters particularly enjoyed a ‘Who wants to be a millionaire’ quiz with questions about physical activity and healthy eating, and a ‘top trumps’ card game (Table 2). They also commented that they enjoyed trying different healthy foods each week. However, it was suggested during the peer supporter focus groups that the follow-up sessions in school were less enjoyable than the off-site training: ‘Um I didn't like them as much as I liked going far away. Because it was like a bit better. Like because we were away from school, like getting out a bit’ (School 24, peer supporter).

The trainers also felt it was more difficult to motivate the peer supporters during the school-based follow-up sessions: ‘There was some taking more interest than others’ (School 21, trainer 6). The school environment constrained the style and content of delivery: ‘Difficult to set-up in time’ (School 22, trainer 1). In addition, limited space and facilities were available for physical activity or food preparation and consumption.

### The cost of the intervention

The total cost of implementing the intervention was £33 866 which equated to an average cost of £11 289 per school. Implementing the intervention in the largest school was the most expensive at £14 964 because the peer supporters were split into two separate groups and additional trainers were required (ratio 1:10 based on ASSIST). The average cost for the two schools where peer supporters were not split into two separate groups was £9451.

### Diffusing the message

Excerpts from the focus groups suggest that some peer supporters modified their own behaviour, or spoke with peers about what they had learned, while others found the role more difficult (Box 1).

Box 1. Focus group responses about message diffusion in the intervention schools

School 22: Peer supporter focus group
  Researcher: Can I just want to check then how easy you thought it was after you‘d had that training to try and give those messages to other people?
  22 032 (female): It wasn't that easy, it really wasn't
  22 099 (female): I, it was a bit
  22 125 (female): It was a bit hard to
School 24: Peer supporter focus group
  Researcher: Does anybody think that going on the training made them change the way they eat?
  24 012 (female): I haven't had a [well known hamburger] since
  24 054 (female): I don't eat [well known hamburger]
  Researcher: And what about [24 097], are you serious that you have changed?
  24 097 (female): Yeah, I eat the dragon fruit! [first tasted during the ‘taste trial’]
School 22: Non-peer supporter focus group
  Researcher: Can you think of anything that you were told about the training or by these people [*peer supporters*]?
  22 156 (male): I'm not sure if it-
  Researcher: Or the information that they learned
  22 156 (male): I'm not sure if it's part of it but people were telling me, you know, having their breakfast and cereal and things like that and just discussing about foods and things like that
School 24: Non-peer supporter focus group
  Researcher: Did they actually speak to you about physical activity or what they'd learnt?
  24 007 (female): No they [*peer supporters*] just said it was fun and everything
  24 030 (male): ‘You should have been there’
  [*Later in the focus group*]
  Researcher: Did they try to encourage you to be more physically active?
  All: No
  24 065 (female): They aren't more physically active either

Responses to several questions in the post-intervention behavioural questionnaires suggest that approximately one-third of students were aware of talking to a peer supporter about physical activity and/or healthy eating, and a similar proportion reported that this had prompted them to increase healthier behaviours. A slightly higher proportion of the year group (38–45%) indicated that they had not had such conversations with a peer supporter, and 11–26% were unsure. However, since peer supporters were asked to work informally they may not have identified themselves as ‘peer supporters’ when having conversations with their friends and peers, and this may have resulted in under-reporting of their activities.

### Potential effectiveness of the intervention

The behavioural questionnaire responses to questions about target food consumption, comparing intervention and control groups at baseline and follow-up, are presented in Table 3. A higher percentage of young people in the intervention group reported consumption of breakfast most days or every day (*χ*^2^ 8.44, *P* = 0.004) and of at least three portions of fruit a day (*χ*^2^ 3.68, *P* = 0.055) than those in the control group. However, consumption of fruit at baseline was already higher in the intervention group. There was no statistically significant difference in the reported consumption of other target foods between the intervention and control schools.

The means and standard deviations for a variety of objective physical activity measures, by trial arm at baseline and follow-up, are presented in Table 4. These show inconsistent and modest differences between intervention and control groups at both time points. Analyses adjusted for baseline physical activity levels, also presented in Table 4, show that mean counts per minute (cpm) were significantly lower and sedentary minutes were significantly higher in the intervention group compared with the control group. There was no difference in MVPA minutes between the intervention and control schools.

**Table 4:** Results: physical activity objective measures and physical activity analysis of covariance

Results: physical activity objective measures		
Physical activity at baseline	Control (*n* = 310) Mean (SD)	Intervention (*n* = 304) Mean (SD)
		
CPM	490.08 (146.59)	496.03 (192.45)
Sedentary minutes per day	464.61 (64.32)	470.23 (65.46)
MVPA minutes per day	38.30 (8.42)	39.02 (8.49)
Physical activity at follow-up	Control (*n* = 211) Mean (SD)	Intervention (*n* = 233) Mean (SD)
CPM	561.76 (177.39)	514.28 (191.36)
Sedentary minutes per day	459.87 (68.68)	473.68 (66.59)
MVPA minutes per day	38.30 (9.30)	37.89 (8.53)

Results: physical activity analysis of covariance*	Mean difference intervention-control (95% CI)	*P*-value
		
CPM	−30.70 (-60.55, -0.85)	0.044
Sedentary minutes per day	14.77 (4.05, 25.49)	0.007
MVPA minutes per day	−0.41 (-1.82, 1.00)	0.569

*Adjusted for baseline physical activity (n = 386).

CPM, counts per minute; MVPA, moderate-to-vigorous physical activity.

## DISCUSSION

The aim of the AHEAD study was to test the feasibility of adapting the ASSIST peer-education model, shown to be effective in reducing smoking uptake, with a view to increasing healthy eating and physical activity amongst adolescents. The primary outcomes of the feasibility study relate to recruitment and retention of schools and young people, the ability to implement the intervention and the acceptability of the intervention. Although not primary outcomes, statistical analyses were undertaken to examine any evidence of promise in relation to physical activity and eating behaviour. We found no clear or consistent evidence of promise, and the data from the process evaluation contribute to our understanding of why this was the case.

Elements of the study were successful. Schools and students were willing to participate and found the AHEAD intervention acceptable. However, two important areas of concern led the research team to conclude that it would not be feasible to implement the AHEAD intervention on a larger scale for evaluation by a full-scale cluster randomised controlled trial. These were the complexity of the messages involved, and; the workload, resources and ensuing costs required to implement the ‘experiential’ training programme.

### The complexity of the health promotion message

Although smoking prevention is not easy, the central message of ASSIST is not to start smoking. AHEAD was based on two key messages: eat healthily and be more active. The focus on both physical activity and healthy eating was deemed necessary since ‘energy balance’ is a key determinant for obesity prevention. Khambalia *et al.* (Khambalia *et al.*, 2012) reported certain intervention components in the school setting as successful in controlling or preventing obesity, one being combined diet and physical activity interventions. However, both messages are multi-dimensional. The trainers indicated that a great deal of background information was required for them to be confident in delivering the training programme and answering young people's queries. It was apparent that to deliver AHEAD required an experienced and confident team of people with expertise in physical activity and healthy eating, and the skills to transmit this information to young people.

However, the ASSIST model does not stop at training young people. The next stage is for the group of trained students, ‘peer supporters’, to diffuse the messages to their peers in their own words in order to effect behavioural change. The dual focus on physical activity and healthy eating appeared too complex for informal diffusion through adolescent peer networks. Furthermore, even if the messages were successfully translated and diffused, it is important to recognize that young people may not be able to implement the required behaviour change. For example, although they may have choice over the snacks and soft drinks they consume, parents may resist changing the weekly food shop on the advice of their children. Incorporating a family component alongside the peer education model could be beneficial, and again, Khambalia *et al.* (Khambalia *et al.*, 2012) found interventions with such a component to be successful. Similarly, schools may not be able to support their students' choice to actively commute by bicycle if their environment lacks the necessary structures and facilities. Therefore, environmental changes may also be necessary.

It may also be the case that ASSIST was successful because what the peer supporters were asked to do, in terms of diffusing anti-smoking messages, was consistent with wider social norms about not smoking. Wider societal norms about physical activity and healthy eating are less supportive of the messages the peer supporters in AHEAD were asked to convey (Ball *et al.*, 2010). For example, Neumark-Sztainer *et al.* (Neumark-Sztainer *et al.*, 1999) reported that we need to change social norms to make it ‘cool’ to eat healthily. Similarly, the obesenogenic environment is extremely unsupportive of attempts to be physically active and eat well (Jackson *et al.*, 2013).

### Experiential learning

The second major challenge in delivering the AHEAD intervention was related to the experiential nature of the training programme. During ASSIST, the key experiential element of the training programme was role-playing conversations with peers. This element was included in AHEAD but the other important experiences involved tasting different foods, preparing food and drinks, playing physical activity games and ‘feeling’ the difference between low, moderate and intense physical activity. These experiences were central to the training but inevitably meant that the intervention was resource and labour intensive, as well as requiring additional attention to venue choice and health and safety issues. Although it proved possible in a relatively small research study, it was felt unlikely that this could be replicated on a much larger scale without increasing the costs considerably.

The resources and the group size required for the AHEAD intervention meant that the costs were almost twice those for ASSIST (£4700 versus £9450 per school). Furthermore, the trainers concluded that no more than 20 peer supporters should be in a training group to enable them to get the most out of the training (ASSIST set the maximum training group size at 30). Limiting the group size in this way would require two separate training groups in an average-sized comprehensive school in England. This would increase the average cost of the intervention by ∼£5500 per school, to about three times the cost of ASSIST, and this would be hard to justify.

## CONCLUSION

While it proved possible to adapt the ASSIST school-based, peer-led smoking prevention intervention to focus on physical activity and healthy eating, to do so was resource and labour intensive and relatively expensive. Limits to peer education also became apparent. If a health promotion message is to be informally diffused through adolescent peer networks it should be relatively simple for trainers to teach and students to pass on. Because the AHEAD intervention focused on two complex behaviours this was not the case. This identifies a tension, particularly for adolescent peer-led health promotion, between the desire not to isolate or oversimplify health behaviours and the need to present clear, succinct health promotion messages.

## FUNDING

The work was undertaken with the support of The Centre for the Development and Evaluation of Complex Interventions for Public Health Improvement (DECIPHer), a UKCRC Public Health Research Centre of Excellence. Funding from the British Heart Foundation, Cancer Research UK, Economic and Social Research Council (RES-590-28-0005), Medical Research Council, the Welsh Government and the Wellcome Trust (WT087640MA), under the auspices of the UK Clinical Research Collaboration, is gratefully acknowledged. Funding to pay the Open Access publication charges for this article was provided by The Wellcome Trust.
